# Development of a web-based intervention for the indicated prevention of depression

**DOI:** 10.1186/1472-6947-13-26

**Published:** 2013-02-20

**Authors:** Saskia M Kelders, Wendy TM Pots, Maarten Jan Oskam, Ernst T Bohlmeijer, Julia EWC van Gemert-Pijnen

**Affiliations:** 1 Faculty of Behavioral Sciences, Department of Psychology, Health and Technology, Center for eHealth Research and Disease Management, University of Twente, PO Box 217, 7500, Enschede, AE, The Netherlands; 2 National Institute for Public Health and the Environment, PO Box 1, 3720, Bilthoven, BA, The Netherlands

**Keywords:** Development, Web-based intervention, Depression, Indicated prevention, Process evaluation, Acceptance and commitment therapy

## Abstract

**Background:**

To reduce the large public health burden of the high prevalence of depression, preventive interventions targeted at people at risk are essential and can be cost-effective. Web-based interventions are able to provide this care, but there is no agreement on how to best develop these applications and often the technology is seen as a given. This seems to be one of the main reasons that web-based interventions do not reach their full potential. The current study describes the development of a web-based intervention for the indicated prevention of depression, employing the CeHRes (Center for eHealth Research and Disease Management) roadmap. The goals are to create a user-friendly application which fits the values of the stakeholders and to evaluate the process of development.

**Methods:**

The employed methods are a literature scan and discussion in the contextual inquiry; interviews, rapid prototyping and a requirement session in the value specification stage; and user-based usability evaluation, expert-based usability inspection and a requirement session in the design stage.

**Results:**

The contextual inquiry indicated that there is a need for easily accessible interventions for the indicated prevention of depression and web-based interventions are seen as potentially meeting this need. The value specification stage yielded expected needs of potential participants, comments on the usefulness of the proposed features and comments on two proposed designs of the web-based intervention. The design stage yielded valuable comments on the system, content and service of the web-based intervention.

**Conclusions:**

Overall, we found that by developing the technology, we successfully (re)designed the system, content and service of the web-based intervention to match the values of stakeholders. This study has shown the importance of a structured development process of a web-based intervention for the indicated prevention of depression because: (1) it allows the development team to clarify the needs that have to be met for the intervention to be of use to the target audience; and (2) it yields feedback on the design of the application that is broader than color and buttons, but encompasses comments on the quality of the service that the application offers.

## Background

To reduce the large public health burden of the high prevalence of depression, preventive interventions targeted at people at risk (indicated prevention) are essential and can be cost-effective [1,2]. However, recruiting participants for interventions to prevent the onset of depressive disorders is quite a challenge [3]. Developing and implementing web-based interventions provide an opportunity to overcome this challenge by tackling the reasons for the low participation rates [3-5]. For example, web-based interventions can decrease the stigma associated with a (mental) health condition by providing a certain degree of anonymity [6,7]. Advantages of web-based interventions can be seen not only in the broader reach, but also in increasing convenience for the users, the opportunity to provide information in an interactive and timely manner and cost-effectiveness [7-9]. Meta-analyses on web-based interventions for the treatment and indicated prevention of mental health complaints have shown that these interventions, on average, are effective in reducing the severity of mental health complaints [10,11].

However, not all web-based interventions show these positive effects. In many cases the effects are less than expected and the implementation of these interventions in regular care is lacking [5,12-14]. It seems that the problem of non-adherence, i.e. participants not following the intervention protocol, is one of the issues behind the lacking effect [15-17]. Studies have shown that better adherence is associated with better (clinical) outcomes of an intervention (see for a systematic review [18]). The reasons behind non-adherence are still unclear, although there are many proposed reasons. Important proposed reasons are: issues with the usability of the application [16]; issues regarding the attunement of the goals of the technology with the aims of the participants [13,19]; and implementation issues as the lack of clarity in the costs-benefit structure and integrating the technology in usual care and daily life [20,21].

Web-based applications are developed at a startling rate, but there is no scientifically underpinned agreement on how to best develop these applications [22]. Many web-based interventions seem to be designed ad hoc; there is a presumed problem for which technology is supposed to be the solution, or the technology is used as a starting point and is developed because of the technological possibility, not because of the needs of the target group. In many cases, the content of these web-based interventions has been the subject of research and consists of evidence-based therapies, but when creating a web-based intervention based on this content, the technology is seen as a given. This ad hoc design and a lack of a holistic overview, in which the human and technological context is given a prominent place, seems to be one of the main reasons that web-based interventions do not reach their full potential in terms of adherence and outcomes [13,22,23].

**Figure 1:**
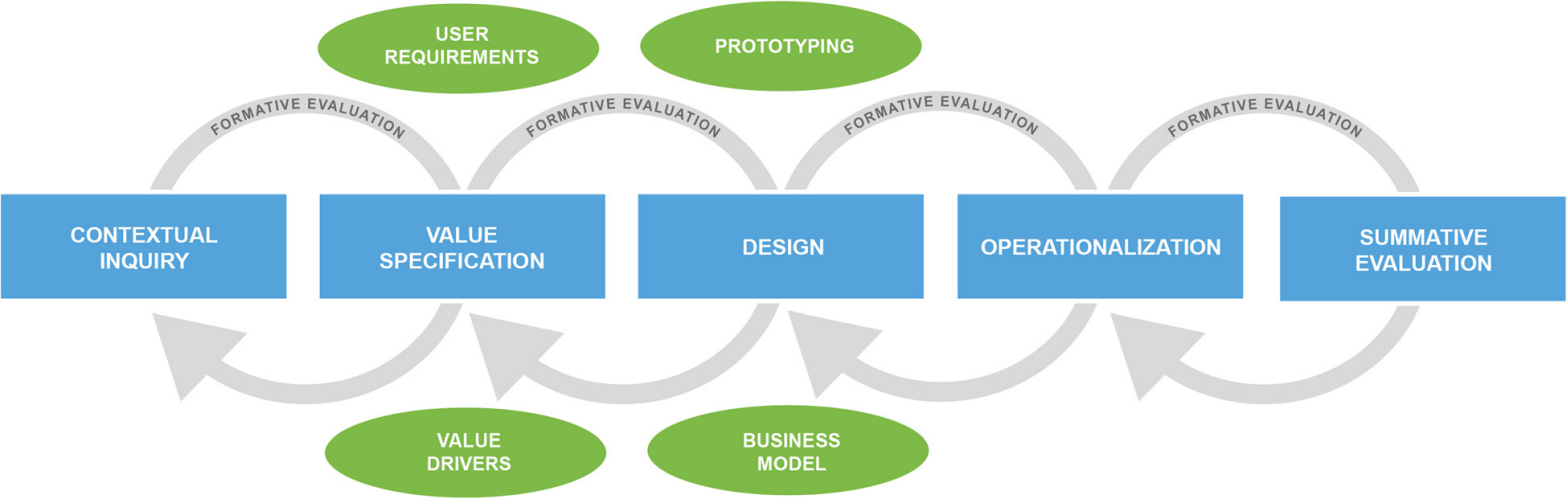
CeHRes roadmap for eHealth development.

In a recent viewpoint paper [13] a holistic framework to improve the uptake and impact of eHealth technologies was proposed. This framework is aimed at overcoming the problems described in the earlier paragraphs. The framework is based on persuasive technology theories, human centered design approaches and business modeling. Persuasive technology refers to the capacity of technology to influence behavior and is used in eHealth research to understand the role of technology in changing behavior [24,25]. Human centered design advocates the systematic, continuous consultation of potential users during the whole design process [26] and has been shown to have a positive effect especially on user satisfaction and on fitting to user needs [27]. Business modeling stems from commercial strategic management [28] and focusses on value creation with stakeholders. In eHealth this approach can be used to make the development of eHealth technology value-driven, i.e. creating technology that matches the values of and makes sense to the different stakeholders [21]. The framework includes the CeHRes (Center for eHealth Research and Disease Management) roadmap for the development of eHealth technologies (Figure 1). This development strategy prescribes a structured development of not only the content of an intervention, but also of the system (or technology) and the service (including the long term implementation). Differences with other development approaches (e.g. Intervention Mapping [29] and the RE-AIM framework [30,31]) lie in the specific focus of the development of the technology and the fit between technology and the content; and in the attention for implementation during the whole process of development. In this paper, we describe a study into the development of a web-based intervention for the indicated prevention of depression, focusing on the first steps of development according to the CeHRes roadmap: contextual inquiry, value specification and design. Important to note is that, although the roadmap describes separate activities with a summative evaluation as the last activity, the roadmap is iterative in nature and prescribes continuous evaluation. This is done through formative cycles that reflect on and inform the development process and check whether the intended goals of the technology are reached. According to the roadmap, each development process should have multidisciplinary project management that facilitates between the creators and the users of the technology. In this study, the project management team consisted of researchers, a clinical psychologist working at the University and at a mental health institute, a developer of the course ‘Living to the full’ (which is the basis for the content of the web-based intervention) who is working at the University, a web-designer and technical programmers.

In the contextual inquiry, information is gathered from the intended users and their environment to see whether there is a need for technology and how this technology might fit into the daily routines of the intended users. In this study, this is done by conducting a literature scan, combined with discussions with the project management team.

The value specification builds on the results of the contextual inquiry and here the key stakeholders determine and rank their values. These values are cooperatively translated into requirements of the technology. In this study, this was done by conduction interviews combined with rapid prototyping [32] with prospective users, and a requirement session with the project development team, consisting of researchers, designers and programmers.

In the design step, (a prototypical version of) the technology is developed, based on the requirements. The framework states that the quality of the design can be assessed at the levels system quality (user friendly application that matches the end-users’ role and task), content quality (providing meaningful and persuasive information) and service quality (providing an adequate and feasible service that fits the context) [33]. In our study, this was done by conducting an expert-based usability inspection, a user-based usability evaluation and a requirement session with the project development team.

The goal of this study is twofold. The first goal is to create a user-friendly application which fits the values of the stakeholders and which can be implemented in daily routine. Our second goal is to evaluate the process of development. The significance of this study is obvious for the application being developed, but additionally, the results regarding the actual application and the process of development can be used as a vantage point when developing similar web-based applications.

In the following sections, of each step of the CeHRes roadmap that we undertook, the method and results will be described. We have chosen to present these sections per step, to increase clarity and to retain the iterative process of the development. The study was conducted between January and March 2010.

## Contextual inquiry

### Methods

To gain a better insight in the context of web-based interventions for the indicated prevention of depression, we performed an exploratory literature scan to gain an overview of the context of our to be developed web-based intervention. Specific goals of the literature scan were: gaining insight in the need for a web-based intervention for the indicated prevention of depression, in features that might enhance the effect of a web-based intervention, and in the effectiveness of the course that provides the content for the web-based intervention. Additionally, we discussed the goals of the project and the needs of project team with the project management team.

### Results

The literature scan revealed a supposed need for easily accessible interventions for the indicated prevention of depression [2,3,5,34-37]. Web-based guided self-help interventions are frequently mentioned as potentially meeting this need [3,5,34,37]. However, this need is mostly stated by care professionals and researchers, for the intended clients.

Our literature scan for features that might enhance the effect of a web-based intervention found strong indications that providing support is essential for an intervention to be effective [38,39]. Furthermore, there is indicative evidence for the effectiveness of using additional text messages to communicate with participants [40]. This is strengthened by the results of a systematic review that shows that periodic prompts can enhance the effectiveness of web-based interventions [41]. Lastly, diaries for self-monitoring have been shown to be successful in a variety of mental health conditions [42] and have been advocated as a useful persuasive technology feature [24,25].

The content of the web-based intervention that will be developed, is based on the self-help book ‘Living to the full’ [43]. This intervention is based on acceptance and commitment therapy (ACT; [44]) and mindfulness [45,46], and targets experiential avoidance that can be considered as a generic risk factor for mental illnesses [47]. Several studies show the effectiveness of this intervention as indicated prevention for depression for a similar target group as is intended for the web-based intervention [48-50]. Studies of Bohlmeijer et al. [48] and Fledderus et al. [50] showed that ‘Living to the full’ as a group course is effective in substantially reducing depressive symptomatology and improving positive mental health in adults. A second study of Fledderus et al. [49] showed that the ACT-based intervention as a self-help programme with both minimal and extensive email support was effective for people with mild to moderate depressive symptomatology.

During the discussion of the goals of our development study and the needs of the project team, we decided that the web-based intervention that will be developed, is, at this stage, primarily a research tool. The reasons for this decision were on the one hand practical: there was no specific implementation setting for which the intervention could be developed (e.g. there was no participating Mental Health Care Organization). On the other hand, this decision was made because the project team wanted to be able to experiment with the web-based intervention, which was decided to be most practical when the intervention would be developed as a research tool. In line with this decision, the most important stakeholder groups were decided on. Because of the choice for a research implementation setting, researchers were deemed an important stakeholder group and, in this stage, care providers were less prominent in the development process. Again, this decision was, in part, a practical decision. The care providers in the research setting were a member of the project team (who was involved) and students of the University who will participate in the counseling of future participants of the intervention, but were not known at the time of the study.

## Value specification

### Methods

In this stage it was determined which values (in this respect, values are anything that a stakeholder deems important related to the goals of the web-based intervention and can be socio-economical as well as behavioral) the prospective users deem important and how they could be implemented in the design of the intervention. This was done by investigating the expected needs of prospective end-users, i.e. people with mild depressive symptoms who were willing to participate in a preventive intervention, using interviews combined with rapid prototyping. Needs are seen as an amplification of the often more abstract values and are expected to be easier voiced by the participants. To translate the expected needs into requirements, a requirement session with the project management team was held.

#### Interviews combined with rapid prototyping

Semi-structured interviews were performed to identify general expected needs of the target group and specific expected needs regarding the usefulness of the features that came forward in the contextual inquiry. The interviews were combined with rapid prototyping [32]. In total, 18 interviews were conducted, which was a similar number of participants used in the study described by Kinzie et al. [32]. The interview participants were people that were interested in participating in a previous study into the effectiveness of ‘Living to the full’ as a guided self-help format with e-mail support [49], but could not enroll in that study because the maximum number of participants was reached. All participants received a gift voucher for their participation. Prior to the interview, the interviewer explained the goal and process of the interview, obtained permission to audio record the interview and each interviewee signed an informed consent. A typical interview lasted about 45 minutes.

**Figure 2:**
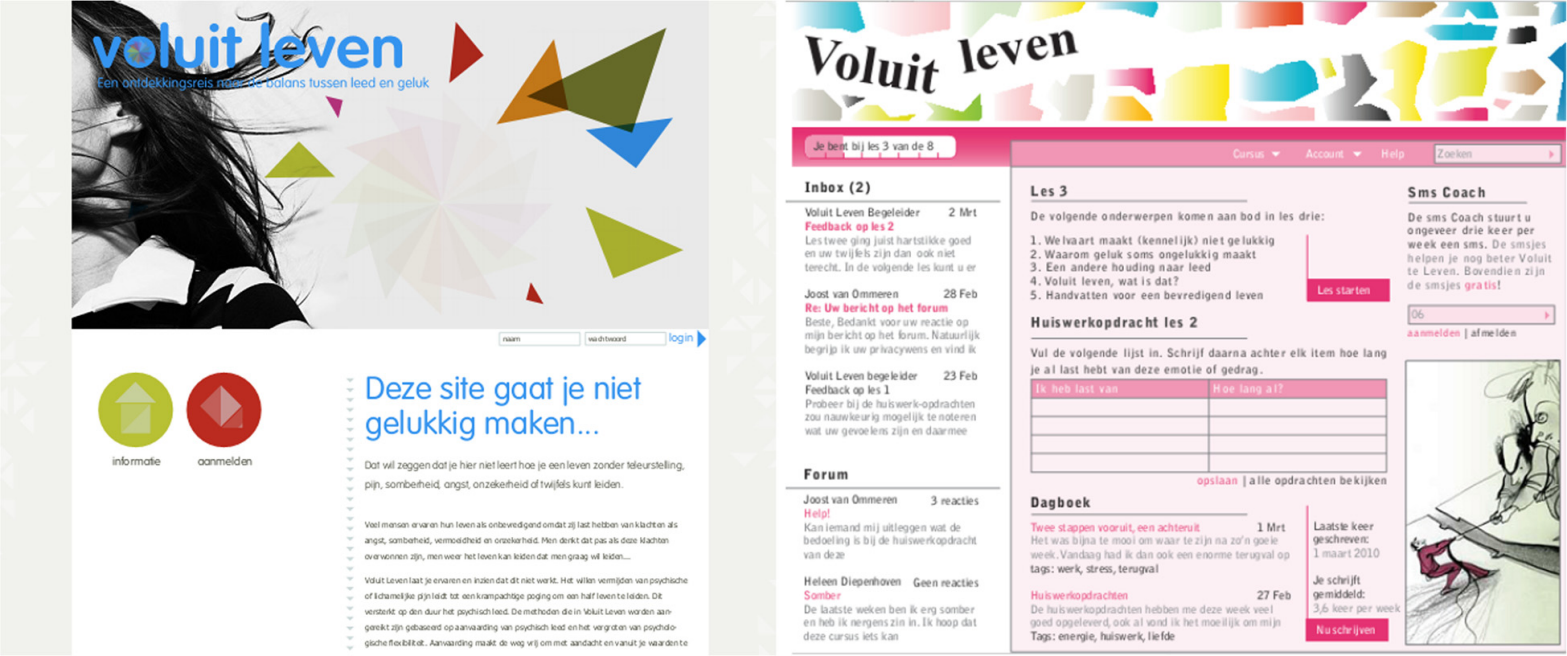
Paper prototypes: general homepage, First design (Left); Personal homepage, Second design (right).

The interview scheme was based upon eHealth and Human Centered Design literature [13,32,51,52] and consisted of three parts. Part one focused on previous experience with the content of the intervention and with web-based interventions in general to assess the background and experience of the participants. Additionally, the expected needs were discussed by asking three cruxes that the web-based intervention had to satisfy and one aspect that would be a reason to quit (or not to start) the intervention. Rapid prototyping was part two of the interview and focused on the usefulness of three features that were available as a paper prototype, i.e. text message coaching, online diary and support in the form of a feedback message. Furthermore, satisfaction with the design and usefulness of the general application was assessed by asking the participants to comment on two different designs of a general home page, a personal homepage and a page with an assignment within the course. The general homepage of the first design and the personal homepage of the second design are presented in Figure 2. All paper prototypes can be found in Additional file 1. Part three assessed demographics, such as age, education and internet experience. The interviews were analyzed within 48 hours after the interview had taken place [53]. We used an inductive thematic analysis to identify patterns in the responses [54]. All analyses were done by two independent coders (SK and MO).

#### Requirement session

The results of the interviews and rapid prototyping were communicated to the designers and programmers by means of a report written by the researchers. The report consisted of the thematic analyses of the responses of the participants. The responses were summarized and quantified where possible, but no interpretation was made by the researchers. During the discussion of the results with the project development team, the responses were translated into requirements. The researchers addressed the expected needs and categorized comments of the participants, and clarified them when they were unclear to the designers and programmers. The designers and programmers used their expertise to fit the expected need or comment in a requirement. The researchers verified whether the requirement did truly meet the expected need or comment by checking the actual comments as they were expressed by the participants.

### Results

#### Participants

The mean age of the 18 participants was 45 years (range 26–62, sd = 10), 78% (n = 14) was female and 78% (n = 14) completed at least higher vocational education. Of the participants 50% (n = 9) had no experience with mindfulness or meditation, 33% (n = 6) had experience with meditation, 6% (n = 1) had experience with mindfulness and 11% (n = 2) had experience with the course ‘Living to the full’. Self-reported experience with using the Internet was high for 39% (n = 7), medium for 56% (n = 10) and low for 6% (n = 1) of the participants. After the interview 78% (n = 14) indicated that they would use the course when available as a web-based intervention, 6% (n = 1) would not use the application and 17% (n = 3) was not sure whether they would use the application or not.

#### Expected needs

The expected needs that were mentioned by the participants were inductively categorized (Table 1; see Additional file 2 for an overview of the specific expected needs). Almost all participants (n = 17) expected a need for professional support and feedback. These needs were formulated as, for example, ‘The system needs to provide contact with a counselor’ (n = 8) or ‘The application needs to provide feedback on your progress’ (n = 8). A need specifically targeted at the system itself was expected by 78% (n = 14). ‘The application needs to be user friendly’ was the most often mentioned need (n = 12). Content needs were expected by 13 participants (72%), where they most often needed the content to have added value (n = 8) and be effective (n = 6). A service need was expected by 56% (n = 10). Service needs related to the process of receiving care through technology, for example, ‘The course needs to have a flexible time planning’ (n = 5) or ‘The course needs to have a fixed endpoint’ (n = 3). Finally, 8 respondents (44%) expected a need for contact with others using the application (peer support).

**Table 1:** Categories of expected needs of prospective users

**Category**	**n**	**%**
Professional support and feedback	17	94
System	14	78
Content	13	72
Service	10	56
Peer support	8	44

#### Usefulness of features

Of the participants, 67% (n = 12) indicated that they would use text message coaching. 11% (n = 2) would not use this feature and for the remaining 22% (n = 4) using this feature would depend on the content of the text messages. Reminders (n = 11) are seen as the most useful content for the text messages, although assignments (n = 6) and motivation (n = 4) were also seen as useful content. The possibility of an online diary was received with mixed reactions. Of the respondents, 44% (n = 8) would definitely use it and saw it as a pleasant addition. However, 56% (n = 10) would only use the diary when it is a part of an assignment in the course. All respondents indicated that support in the form of feedback messages would be useful and essential. Feedback is expected on assignments, but furthermore, it could be useful for gaining new insights, support and motivation. Several respondents (n = 5) stress the importance of the feedback being personal.

#### Feedback on design

For both the homepage and the exercise, the first paper prototype was preferred (56% and 77% respectively). Positive associations with the first prototype were ‘professional’ (n = 5), ‘calm’ (n = 5) and ‘clear’ (n = 3). Negative associations were less common, but ‘bleak’ was mentioned by 2 respondents. For the second prototype the most mentioned association was ‘busy’ (n = 5), followed by ‘cheerful’ (n = 4). Furthermore, comments of the participants on both paper prototypes regarded the appropriateness and attractiveness of the used images, shapes, header, color, font, text, buttons, menu structure and process indicator. An example of the written comments of two participants on two paper prototypes is presented in Additional file 3.

#### Requirement session

The results of the interviews were discussed with the project development team. The expected needs of the participants that were not yet met in the design of the web-based intervention, were discussed regarding urgency, feasibility and desirability according to the stakeholders (participants, researchers, designers and programmers). This resulted in requirements regarding the system, the service and peer support, where for example for peer support, it was chosen not to fully incorporate the need of the participants for ‘contact with others using the application’ but to include prewritten experiences of people who used the self-help book ‘Living to the full’. The comments of participants regarding the appropriateness and attractiveness of the used images, shapes, header, color, font, text, buttons, menu structure and process indicator were discussed and translated into requirements that were implemented in the prototype. Finally, a time planning for building the prototype was agreed upon, including another requirement session where the progress was discussed.

## Design

### Methods

A working prototype of the web-based intervention was developed according to the requirements specified in the value specification stage. The prototype consisted of the registration procedure, the first lesson of the course and the personal home page with the features: testimonials, diary, text message coach, overview of completed assignments and overview of feedback. The prototype was evaluated on system quality, content quality and service quality by conducting an expert-based usability inspection and a user-based usability evaluation. In line with the recommendation by Jaspers [55], we have chosen to employ both a user-based and an expert-based evaluation method. We have investigated issues identified by these methods and the overlap of identified issues by both methods. This knowledge will help us make founded choices on which methods to use in similar development processes. Issues that came forward using these evaluation methods were combined into points for improvement, which were discussed with the development team to translate these points into requirements and prioritize these requirements.

#### User-based usability evaluation

We employed a scenario-based think aloud protocol [55], i.e. prospective users were guided through the application by means of scenarios that pose a problem or task that may be solved or completed by using the program, and respondents were instructed to verbalize their thoughts during the whole test. We conducted usability tests with ten prospective users, which is deemed sufficient to identify the main usability problems [56]. Participants were recruited using online advertisements and were part of the target group of the web-based intervention. All participants received a gift voucher for their participation. Prior to the usability test each participant signed an informed consent. All usability tests were recorded and coded retrospectively. The material (audio and video) was reviewed and comments were identified by the researcher. Comments were defined as relevant verbalizations of a thought, problems encountered by the participants, tasks that were completed smoothly and relevant feedback the participant provided during the interview. These comments were analyzed using a coding scheme following the work of DeLone and McLean [33] and Van Gemert-Pijnen [13] which distinguishes between system quality, content quality and service quality. System quality refers to the user friendliness of the application, including the placement of buttons and the lay-out of the application. Content quality refers to the usefulness and persuasiveness of the information presented in the application, including spelling and understandability of all texts in the application. Service quality refers to the process of care given by the application, including the registration procedure and features that have (not) been included.

#### Expert-based usability inspection

The cognitive walkthrough method [55] was used to assess the usability of the application by experts. In a cognitive walkthrough, experts analyze and evaluate the steps a typical user would take when trying to reach a certain goal. Important in a cognitive walkthrough is that it is specifically guided by user tasks or goals. The experts that carried out the cognitive walkthrough were all eHealth researchers and were working at the University of Twente. One of the experts is a clinical psychologist and has expert knowledge about the target group. Issues were coded using the same coding scheme as used for the user-based usability evaluation. To check for differences in coding, 20% of the results from the user-based usability evaluation and 20% of the results of the expert-based usability inspection were coded by two researchers (SK and JvG). The interrater reliability, measured by Cohen’s kappa, was 0.84 for the categories ‘content’, ‘system’ and ‘service’, and 0.90 for the categories ‘positive’, ‘neutral’ and ‘negative’.

#### Requirement session

The report written by the researchers contained the points for improvement which were the summarized comments from both evaluation methods. The points for improvement were clustered along different parts of the prototype. For each point, the non-interpreted issue was present, but in some cases, a recommendation was added by the researcher, based on the results of the evaluation methods. During the discussion of the results with the project development team, the points for improvement were translated into requirements. The researchers addressed the points for improvement and clarified them when they were unclear to the designers and programmers. The designers and programmers used their expertise to fit the point for improvement in a requirement. The researchers checked whether the requirement did truly meet the point for improvement as it was expressed by the participants. Finally, a prioritization was made considering on the one hand the frequency and urgency of an issue and on the other hand the prediction of time and effort to implement the new requirement.

### Results

#### Participants

The mean age of the 10 participants of the usability test was 38 years (range 24–53, sd = 11), 90% (n = 9) was female and 70% (n = 7) completed at least higher vocational education. The cognitive walkthrough was carried out by 8 participants, who can be categorized as usability experts (n = 7) and a target group expert (n = 1) [55].

#### Evaluation of system, content and service quality

In total, both methods yielded 476 comments, virtually equally distributed between the user-based and expert-based evaluation method (respectively 52% (n = 246) and 48% (n = 230)). Table 2 shows the distribution of the comments over system, content and service quality and the amount of positive (+), neutral (+/−) and negative (−) comments.

**Table 2:** Number of comments yielded from user-based and expert-based methods

	**Users**				**Experts**				**Total**
	**+**	**+/−**	**-**	**total**	**+**	**+/−**	**-**	**total**	
System	50	7	98	155	26	2	99	127	282
Content	18	7	35	60	7	2	57	66	126
Service	9	2	20	31	2	3	34	39	70
Total	77	16	153	246	35	7	190	232	478

Chi square analyses show that there were no significant differences in the distribution of comments on system, content and service between both evaluation methods (χ^2^ = 3.57; p = .168). There was a significant difference in the distribution of positive, neutral and negative comments between both evaluation methods (χ^2^ = 22.9; p < .001), where the user-based method yielded relatively more positive comments and the expert-based method yielded relatively more negative comments.

The subject of positive comments was similar between users and experts. Positive comments on the content were mainly on the texts, which were evaluated as recognizable and easy to read, as well as on the exercises (e.g. the audio mindfulness exercise and choosing a picture that represents your motto and placing this on the homepage), which were deemed fun and useful. Positive comments on the system were mainly on the ease of use of the application (e.g. easy to find specific features, no explanation required) and on the appearance (e.g. fresh, pleasant). Positive comments on the service were mainly on features that were included as the possibility to personalize the homepage through the exercises and text message coaching. Neutral comments were infrequent and were made on, for example, the content of texts and the procedure of doing exercises. All negative comments were clustered on subject and transformed into points for improvement, where multiple comments on the same subject were combined in one point for improvement. The points for improvement were clustered along different parts of the prototype. An overview of points for improvements that arose from the user-based method, the expert-based method and both methods can be found in Table 3.

**Table 3:** Points for improvement from user-based evaluation method, expert-based evaluation method and both evaluation methods

	**Users**	**Experts**	**Both**	**Total**
Registration	2	16	11	29
Textual	2	8	4	14
Lesson 1	2	5	6	13
Cockpit	3	3	5	11
Exercise 1 (‘Backpack with suffering’)	1	2	5	8
Exercise 2 (‘Choose your motto and picture’)	3	1	3	7
Diary	2	5	0	7
Login	0	3	1	4
My data	2	1	1	4
Overview of exercises	0	3	1	4
Exercise 3 (‘Audio: Bodyscan’)	1	2	0	3
Other	0	5	1	6
Total	18	55	38	110

Of the points for improvement, 49% came forward in both methods, 16% came forward only in the user-based method and 35% came forward only in the expert-based method. Most points for improvement were found in the registration (26%). Many of these were on the content (e.g. be consistent in answering categories, the answering categories for ‘most important complaint’ are too limited, some questions are unclear) and layout (e.g. reduce the amount of scrolling needed, align all answering categories). Furthermore, many textual and spelling points for improvement were found (13%). Lesson 1 and the cockpit also received a substantial number of points for improvement (12% and 10%, respectively). In the lesson, many layout improvements were deemed necessary (e.g. the text is too small, the line distance and alignment is not consistent). Furthermore, the procedure could be improved (e.g. it is a lot of reading before the lesson becomes interactive, the table of contents doesn’t completely reflect the contents). On the cockpit, there were improvements needed on labeling (e.g. the ‘cockpit’ needs to be renamed or introduced, ‘chapter’ should be ‘lesson’) and in the system (e.g. the ‘back’ button of the browser needs to function correctly within the application, exercise 1 remains visible when the user returns to the cockpit). The number of points for improvement on the other parts ranged from three to eight and covered issues as the unwanted display of an error message when not selecting an image for your motto (Exercise 2), a lack of instruction for the diary and ‘the lay-out differs between browsers’ (Other).

#### Requirement session

The points for improvement were discussed with the project development team, consisting of researchers, designers and programmers. Requirements and suggestions to meet the points for improvement were formulated. It was decided to build the full web-based intervention based on these requirements and suggestions. Furthermore, a detailed planning was made of when which parts of the intervention would be ready and would be available for error-checking. Additionally, different points for improvement of service quality were discussed with the project management team (including a care provider and a developer of the course ‘Living to the full’) to reach a decision on how to implement these points for improvement. This was done, for example, for ‘be clearer to participants on when telephone screening is needed for the registration procedure’ and ‘is it always necessary that an exercise is completed before a participant can go to the next page?’.

## Discussion

This study was aimed at creating a user-friendly application which fits the values of the involved stakeholders and which can be implemented in daily routine, and at evaluating the process of development. Regarding the application, we can say that the holistic view, incorporating system, content and service as well as the perspectives and values of the different stakeholders provided the opportunity to investigate and develop the technology not as merely a tool, but as an essential part of the care it is intended to provide. The methods (user-based and expert-based) used in this study seem to provide valuable feedback that reaches further than only comments on color or the lay-out of buttons; rather it encompasses the context of the intervention. An important advantage of the user-based methods proved to be that the target audience assesses the prototype from their view and context. The experts seem to be successful in assessing the prototype from the point of view of a participant (hence the large overlap between results from the user-based and expert-based methods), but also seemed to have a more comprehensive view based on their experience. This strengthens the recommendation from literature (e.g. [55]) to involve both experts and users in the development of web-based interventions.

The overall process of development was satisfactory. The combination of iterative stages provided more insights in the goals and processes of the technology we were developing than the separate stages. Each step yielded insights that build on the knowledge from earlier steps and shaped the next step. It must be noted that the methods and steps we have described are not truly separate, but can be viewed as continuous. This entails that the results of the methods used in a step, can be used as input for different steps. For example, the interviews held in the value specification step, have also provided information on the context, which would, ideally be gathered in the contextual inquiry step.

### Limitations

A limitation of this study is that it did not involve all stakeholders in all stages. The users were not involved in the contextual inquiry, while the CeHRes roadmap advocates this. We did conduct a literature scan, but we have not assessed the actual need for a web-based intervention of the target audience. We have tried to overcome this limitation by addressing the needs and expectations of the target audience in the value specification phase. Furthermore, care providers have not been included as stakeholder in this study. This decision was made in the contextual inquiry where we decided to take research as the implementation setting. This choice was not ideal, because research is only an intermediate implementation setting, not a final, viable, long term setting. We have coped with this limitation by including a care provider in the project management team, but nonetheless, when developing this application further for a different implementation setting, care providers need to be involved.

Another limitation is that the target users that were involved in this study were mainly highly educated women with almost half of these (46%) between the ages of 40 and 49 years old. This seems a very specific group for an indicated prevention intervention for which no specific target group is formulated and therefore the results may not be generalizable to other web-based interventions. However, the group that participated in this study is the group that is reached by many web-based interventions (e.g. [16,19,57]) and therefore seems appropriate for this specific web-based intervention. However, when a web-based intervention is intended for other groups, such as men or young people, attention should be paid to involve these people in the development of that intervention.

A further limitation regarding the participants in this study is that all methods have been conducted with small groups of participants and may therefore not be generalizable. However, the number of participants in all methods match the recommended numbers (see [32,55,56]). We feel that the results of this study can be of value to others who are developing a web-based intervention by taking our results as a vantage point. Especially for an intervention targeted at the same audience it is reasonable to believe that the expected needs are similar. Additionally, it may well be that the values we identified are generalizable to the target audience of other web-based interventions as the users of these interventions are most often similar (overrepresentation of highly educated females (e.g. [49]). However, our results should only be taken as a starting point and should be verified in the target audience of the intervention. This iterative process is a core concept in the CeHRes roadmap [13] and covers not only the expected needs and usefulness of certain features, but also the effectiveness of the intervention. To assess the effectiveness of the intervention, it is necessary not only to look at the effectiveness of the intervention as a whole, but also to investigate the active ingredients of the intervention [58].

We have not assessed whether developing a web-based intervention using the CeHRes roadmap is better than developing a web-based intervention in a different way. In our view, this will always remain an issue, because it seems difficult, if not impossible or undesirable, to develop two web-based interventions using different methods, but using the same ideas or content as a starting point. However, we can say that using the methods in this study, we have been able to clarify expected needs for this web-based intervention and we have been able to adapt the intervention to these needs. Additionally, in this study we have not assessed the effectiveness or impact of the intervention. Therefore, we cannot say whether the intervention is successful in reducing depressive symptoms in the target group of the intervention. Future research will need to assess whether the target users will actually use the intervention and whether it has the intended effects. To gain information on how to redesign or refine the web-based intervention to better reach these effects, it is important to employ methods that objectively measure usage, yield qualitative feedback on the satisfaction of users and assess the (clinical) effectiveness.

## Conclusion

This study has shown the importance of a structured development process of a web-based intervention for the indicated prevention of depression because: (1) it allows the development team to clarify the needs that have to be met for the intervention to be of use to the target audience; and (2) it yields feedback on the design of the application that is broader than color and buttons, but encompasses comments on the quality of the service that the application offers. In this study, specific examples of what the structured development process has generated are: more attention to the process and the flow of participants in the application (what do the participants exactly have to do in each lesson, when can they proceed to the next lesson, when do they get reminders etc.); prevented us from creating a complex menu structure in which the users would have lost their way as they indicated in the rapid prototyping stage; the idea that text message coaching can not only be used for reminding participants, but also act as a short assignment and as motivation. Overall, by developing the technology, not only technical aspects are developed, but the whole process, including system, content and service is (re)designed to match the values of stakeholders.

## Competing interests

The authors declare that they have no competing interests.

## Authors’ contributions

SK was involved in phases of the study. WP, MJO, EB and JGP participated in the design and coordination and helped to draft the manuscript. All authors read and approved the final manuscript.

## Pre-publication history

The pre-publication history for this paper can be accessed here:

http://www.biomedcentral.com/1472-6947/13/26/prepub

## Supplementary Material

Additional file 1 ** general homepage, exercise and menu overlay, first design; general homepage, personal homepage and exercise, second design.** Description: Images of all the used paper prototypes. Click here for file

Additional file 2 **Overview of expected needs of prospective users.** Description: Table of all the mentioned expected needs of the prospective users. Click here for file

Additional file 3 **Example of written comments of two participants on two paper prototypes.** Description: Images of two paper prototypes with comments of two participants. Click here for file
