# Supplementary material for: Development of a web-based intervention for the indicated prevention of depression
Source: BMC Med Inform Decis Mak. 2013 Feb 20;13:26. doi: 10.1186/1472-6947-13-26 (PMC3598782; doi:10.1186/1472-6947-13-26)
Supplement: Additional file 1 — general homepage, exercise and menu overlay, first design; general homepage, personal homepage and exercise, second design. Description: Images of all the used paper prototypes. [file 1472-6947-13-26-S1.pdf]

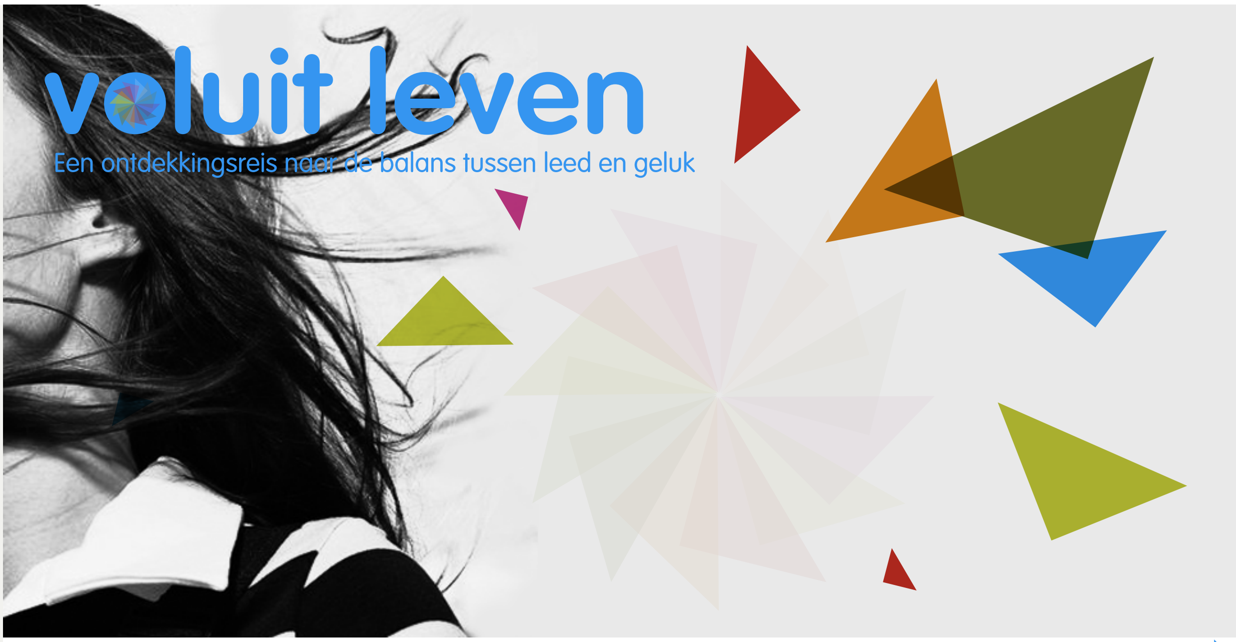

naam | wachtwoord | login

informatie

aanmelden

Deze site gaat je niet gelukkig maken...

Dat wil zeggen dat je hier niet leert hoe je een leven zonder teleurstelling, pijn, somberheid, angst, onzekerheid of twijfels kunt leiden.

Veel mensen ervaren hun leven als onbevredigend omdat zij last hebben van klachten als angst, somberheid, vermoeidheid en onzekerheid. Men denkt dat pas als deze klachten overwonnen zijn, men weer het leven kan leiden dat men graag wil leiden...

Voluit Leven laat je ervaren en inzien dat dit niet werkt. Het willen vermijden van psychische of lichamelijke pijn leidt tot een krampachtige poging om een half leven te leiden. Dit versterkt op den duur het psychisch leed. De methoden die in Voluit Leven worden aangereikt zijn gebaseerd op aanvaarding van psychisch leed en het vergroten van psychologische flexibiliteit. Aanvaarding maakt de weg vrij om met aandacht en vanuit je waarden te leven. Wanneer dit lukt ervaar je wel met de dag meer vrijheid en kwaliteit in je leven...

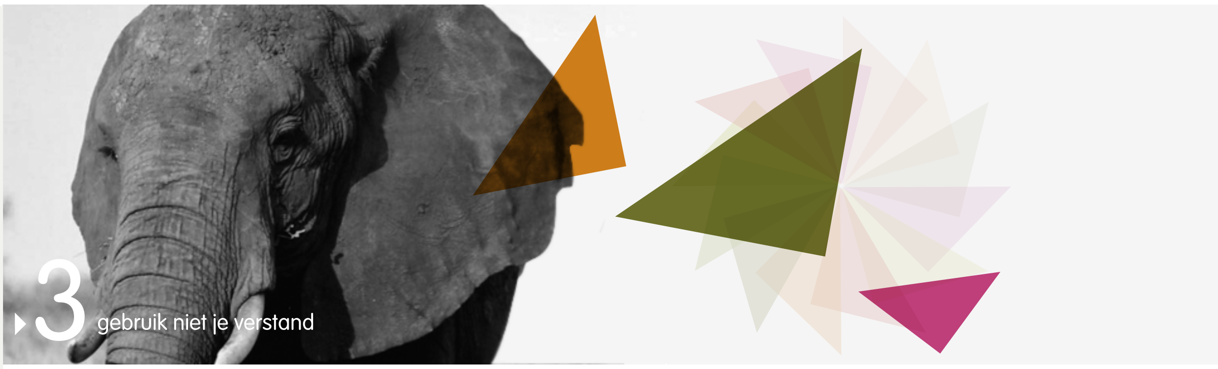

Oefening: de rugzak met leed

In deze oefening willen we je vragen om een overzicht te maken van alles waar je last van hebt en waar je nu mee worstelt in het leven. Het gaat om emoties, gevoelens, gedachten, gewoonten en gedrag waar je vanaf wilt. Het is als het ware de pijnlijke bagage die je dagelijks met je meedraagt in een rugzak. Het is het leed waar je onder gebukt gaat. We geven een aantal voorbeelden:

- Verdriet over mijn overleden kat.
- Ik ben snel boos.
- Ik eet te veel.
- Ik zie elke ochtend op tegen de dag.
- Mijn schuldgevoelens dat ik te weinig tijd heb voor mijn kinderen.
- De gedachte dat ik niet veel waard ben.
- Het gevoel van uitputting.
- Mijn onzekerheid.
- Dat ik te veel drink.
- Etc. etc.

Ik heb last van:

opslaan

< 1 jaar
1 jaar
2 jaar
3 jaar
4 jaar
5 jaar
> 5 jaar

Ik irriteer me aan mijn buren	al 3 jaar
Ik twijfel regelmatig of ik het leven dat ik leid wel zo leuk vind...	al 2 jaar
Ik snoep teveel	al 3 jaar

Zet door middel van de blauwe pijltjes waar je het meeste last van hebt bovenaanen onderaan waar je het minste last van hebt. Wanneer je dit hebt gedaan, kun je erachter zetten in welke mate het gevoel, de gedachte, het gedrag je belemmert om het leven te leiden dat je graag zou willen leiden.
Deze lijst is waarschijnlijk een belangrijke reden waarom je dit boek hebt gekocht en aan de slag bent gegaan. Je hebt al het psychisch leed uit je rugzak gehaald en voor je neer gezet. Het is de stand van zaken van dit moment voordat je aan dit boek bent begonnen. En was het nu maar zo simpel dat je deze bagage naast je neer kon zetten en gewoon weg kon lopen Dan zou dit een dun boekje zijn! Maar de rugzak met psychisch leed zit aan ons vastgeklonken. En de pijnlijke items floepen er vanzelf weer in. We gaan ermee naar bed en staan ermee op. Waarschijnlijk heb je het gevoel dat het deze rugzak is of een aantal items in de rugzak die jou belemmeren in je bewegingsvrijheid, die jou remmen om voluit te leven. In de komende twee weken zul je gaan onderzoeken hoe je met dit leed omgaat. En aan het einde van dit boek zou er iets in de lijst veranderd moeten zijn. We zullen dat in de laatste bijeenkomst nagaan.

Verder

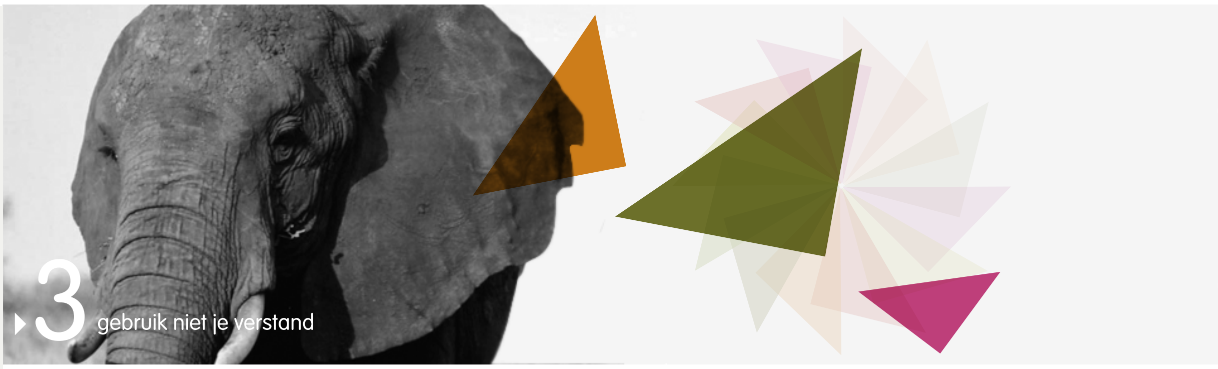

Hans Houkes

Dinsdag, 11 maart 2010 20:10

Nieuw bericht

opslaan

dag week vandaag zoeken

12:30 Twijfels — zichtbaar

Vandaag vond ik het moeilijk om op gang te komen. Ik zag het nut van al deze oefeningen even niet meer zitten. Pas toen ik de vorige oefeningen even door had gelezen en direct een E-mail terug kreeg op mijn hulproep aan een van de studenten wist ik weer waar het om ging: mezelf!. Waarom heb ik steeds het idee dat ik het goed moet doen, dat ik dit hele traject voor iemand anders doe? Ik hoop hier echt een antwoord op te vinden...

09:00 Een nieuwe start — onzichtbaar

Pfff, fijn dat de ochtend begonnen is. Ik lag al vanaf 7:00 te woelen in mijn bed, te wachten tot ik op mocht staan. Van wie ook al weer? Waarom ga ik met mijn slapeloosheid niet om 5:00 met mijn laarzen aan door het bos banjeren? Wat mag ik van wie allemaal niet en waarom? Ik krijg het donker-bruin vermoeden dat alle pijlen terug-wijzen naar me zelf. Ingewikkeld...

et leed

e vragen om een overzicht te maken van
n waar je nu mee worstelt in het leven. Het
s, gedachten, gewoonten en gedrag waar je
re de pijnlijke bagage die je dagelijks met je
aar je onder gebukt gaat. We geven een

or mijn kinderen.

opslaan

< 1 jaar
1 jaar
2 jaar
3 jaar
4 jaar
5 jaar
> 5 jaar

	al 3 jaar
Ik twijfel regelmatig of ik het leven dat ik leid wel zo leuk vind...	al 2 jaar
Ik snoep teveel	al 3 jaar

Zet door middel van de blauwe pijltjes waar je het meeste last van hebt bovenaanen onderaan waar je het minste last van hebt. Wanneer je dit hebt gedaan, kun je erachter zetten in welke mate het gevoel, de gedachte, het gedrag je belemmert om het leven te leiden dat je graag zou willen leiden.

Deze lijst is waarschijnlijk een belangrijke reden waarom je dit boek hebt gekocht en aan de slag bent gegaan. Je hebt al het psychisch leed uit je rugzak gehaald en voor je neer gezet. Het is de stand van zaken van dit moment voordat je aan dit boek bent begonnen. En was het nu maar zo simpel dat je deze bagage naast je neer kon zetten en gewoon weg kon lopen Dan zou dit een dun boekje zijn! Maar de rugzak met psychisch leed zit aan ons vastgeklonken. En de pijnlijke items floepen er vanzelf weer in. We gaan ermee naar bed en staan ermee op. Waarschijnlijk heb je het gevoel dat het deze rugzak is of een aantal items in de rugzak die jou belemmeren in je bewegingsvrijheid, die jou remmen om voluit te leven. In de komende twee weken zul je gaan onderzoeken hoe je met dit leed omgaat. En aan het einde van dit boek zou er iets in de lijst veranderd moeten zijn. We zullen dat in de laatste bijeenkomst nagaan.

Verder

voluitleven.info |

http://www.voluitleven.info/

Google

voluitleven.info |

Voluit leven

- Voluit Leven
- Online cursus
- Ervaringen
- Contact

Wanneer kijk je terug op je dag en weet je dat het klopt? Dat het zo moest zijn? In welke mate ben je je bewust van je reacties op je leven? Dát is Voluit Leven.

Kunnen genieten van het leven en het leven kunnen lijden, zonder lijdzaam te zijn. Handelen vanuit je waarden en weten waar je zelf de stroom van het leven tegenhoudt.

De online cursus Voluit Leven is gratis en bedoeld voor volwassenen met (niet-ernstige) psychische klachten als angst, stress, vermoeidheid, depressiviteit, onzekerheid, verslavingen.

Meld je direct aan voor de online cursus Voluit Leven

Naam

E-mailadres

Wachtwoord

Wachtwoord nogmaals ter bevestiging

Direct aanmelden

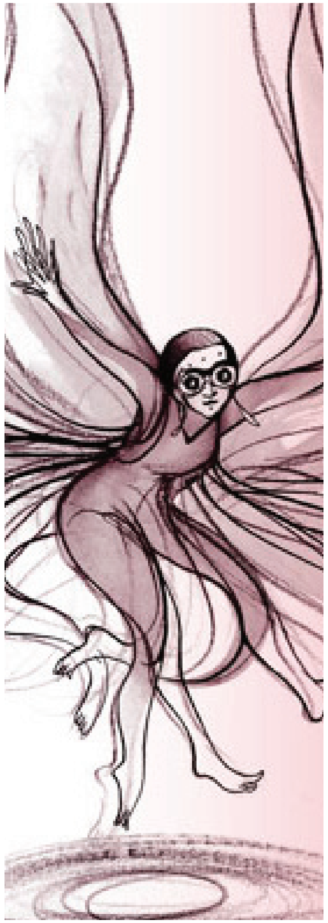

voluitleven.info |

http://www.voluitleven.info/

Google

voluitleven.info |

Voluit leven

Je bent bij les 3 van de 8

Cursus Account Help Zoeken

Inbox (2)

Voluit Leven Begeleider 2 Mrt
Feedback op les 2
Les twee ging juist hartstikke goed en uw twijfels zijn dan ook niet terecht. In de volgende les kunt u er

Joost van Ommeren 28 Feb
Re: Uw bericht op het forum
Beste, Bedankt voor uw reactie op mijn bericht op het forum. Natuurlijk begrijp ik uw privacywens en vind ik

Voluit Leven begeleider 23 Feb
Feedback op les 1
Probeer bij de huiswerk-opdrachten zou nauwkeurig mogelijk te noteren wat uw gevoelens zijn en daarmee

Forum

Joost van Ommeren 3 reacties
Help!
Kan iemand mij uitleggen wat de bedoeling is bij de huiswerkopdracht van deze

Heleen Diepenhoven Geen reacties
Somber
De laatste weken ben ik erg somber en heb ik nergens zin in. Ik hoop dat deze cursus iets kan

Les 3

De volgende onderwerpen komen aan bod in les drie:

1. Welvaart maakt (kennelijk) niet gelukkig
2. Waarom geluk soms ongelukkig maakt
3. Een andere houding naar leed
4. Voluit leven, wat is dat?
5. Handvatten voor een bevredigend leven

Les starten

Huiswerkopdracht les 2

Vul de volgende lijst in. Schrijf daarna achter elk item hoe lang je al last hebt van deze emotie of gedrag.

Ik heb last van	Hoe lang al?

opslaan | alle opdrachten bekijken

Dagboek

Twee stappen vooruit, een achteruit 1 Mrt
Het was bijna te mooi om waar te zijn na zo'n goeie week. Vandaag had ik dan ook een enorme terugval op
tags: werk, stress, terugval

Huiswerkopdrachten 27 Feb
De huiswerkopdrachten hebben me deze week veel goed opgeleverd, ook al vond ik het moeilijk om mijn
Tags: energie, huiswerk, liefde

Laatste keer geschreven:
1 maart 2010

Je schrijft gemiddeld:
3,6 keer per week

Nu schrijven

Sms Coach

De sms Coach stuurt u ongeveer drie keer per week een sms. De smsjes helpen je nog beter Voluit te Leven. Bovendien zijn de smsjes gratis!

06

aanmelden | afmelden

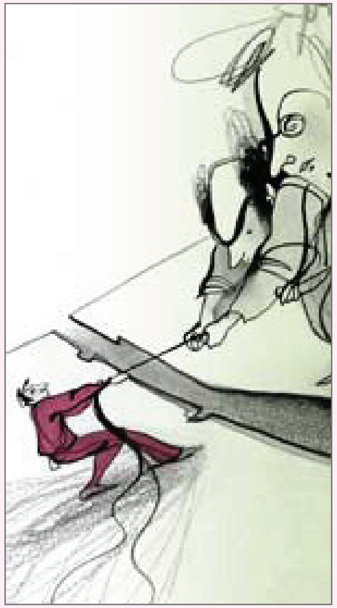

voluitleven.info |

http://www.voluitleven.info/

voluitleven.info |

Voluit leven

63% van les 3 voltooid

Home > Cursus > Les 3 > oefening   Cursus   Account   Help   Zoeken

Inhoud les 3

- Inleiding
- Dagboek
- Oefening
- Metafoor
- Intermezzo
- Mindfulness
- Huiswerkopdracht

- Persoonlijke homepagina
- Dagboek
- Inbox (2)

Oefening: de rugzak met leed

In deze oefening willen we je vragen om een overzicht te maken van alles waar je last van hebt en waar je nu mee worstelt in het leven. Het gaat om emoties, gevoelens, gedachten, gewoonten en gedrag waar je vanaf wilt. Het is als het ware de pijnlijke bagage die je dagelijks met je meedraagt in een rugzak. Het is het leed waar je onder gebukt gaat. We geven een aantal voorbeelden:

- Verdriet over mijn overleden kat.
- Ik ben snel boos.
- Ik eet te veel.
- Ik zie elke ochtend op tegen de dag.
- Mijn schuldgevoelens dat ik te weinig tijd heb voor mijn kinderen.
- De gedachte dat ik niet veel waard ben.
- Het gevoel van uitputting.
- Mijn onzekerheid.
- Dat ik te veel drink.
- Etc. etc.

Ik heb last van	Hoe lang al?

Je hebt al het psychisch leed uit je rugzak gehaald en voor je neer gezet. Het is de stand van zaken van dit moment voordat je aan deze cursus bent begonnen. En was het nu maar zo simpel dat je deze bagage naast je neer kon zetten en gewoon weg kon lopen Dan zou

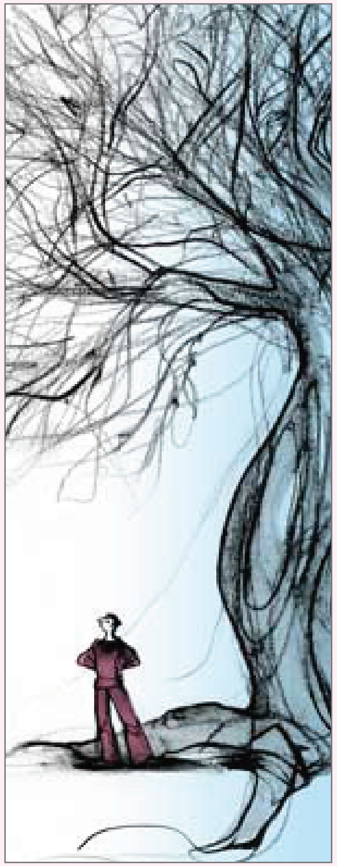

Vorige stap | Volgende stap
