# Supplementary material for: Development of a web-based intervention for the indicated prevention of depression
Source: BMC Med Inform Decis Mak. 2013 Feb 20;13:26. doi: 10.1186/1472-6947-13-26 (PMC3598782; doi:10.1186/1472-6947-13-26)
Supplement: Additional file 2 — Overview of expected needs of prospective users. Description: Table of all the mentioned expected needs of the prospective users. [file 1472-6947-13-26-S2.pdf]

Additional file 2 – Overview of Expected Needs of Prospective Users

Expected Needs	n	%
Support and feedback	*17*	*94*
Contact with counselor	8	44
Feedback	8	44
Personal attention	3	17
Specific instructions	3	17
Chat function	1	6
Being taken seriously	1	6
Application	*14*	*78*
User friendly	12	67
Attractive	5	28
Encouragement to complete the course	3	17
Content	*13*	*72*
Added value	8	44
Effective	6	33
To-the-point	3	17
Focus on real world	1	6
Varied assignments	1	6
Service	*10*	*56*
Flexible time planning	5	28
Fixed endpoint	3	17
Limited time behind pc	1	6
No need to go outdoors	1	6
Flexible pace	1	6
Anonymity	1	6
Combines well with other activities	1	6
Aftercare	1	6
Social support	*8*	*44*
Contact with others using the application	8	44
