# Supplementary material for: Development of a web-based intervention for the indicated prevention of depression
Source: BMC Med Inform Decis Mak. 2013 Feb 20;13:26. doi: 10.1186/1472-6947-13-26 (PMC3598782; doi:10.1186/1472-6947-13-26)
Supplement: Additional file 3 — Example of written comments of two participants on two paper prototypes. Description: Images of two paper prototypes with comments of two participants. [file 1472-6947-13-26-S3.pdf]

voluitleven.info |

http://www.voluitleven.info/

voluitleven.info

Voluit leven

63% van les 3 voltooid

Home > Cursus > Les 3 > oefening   Cursus   Account   Help   Zoeken

mooi overzicht

Inhoud les 3

- Inleiding
- Dagboek
- Oefening
- Metafoor
- Intermezzo
- Mindfulness
- Huiswerkopdracht

- Persoonlijke homepagina
- Dagboek
- Inbox (2)

te veel een boek

Oefening: de rugzak met leed

In deze oefening willen we je vragen om een overzicht te maken van alles waar je last van hebt en waar je nu mee worstelt in het leven. Het gaat om emoties, gevoelens, gedachten, gewoonten en gedrag waar je vanaf wilt. Het is als het ware de pijnlijke bagage die je dagelijks met je meedraagt in een rugzak. Het is het leed waar je onder gebukt gaat. We geven een aantal voorbeelden:

- Verdriet over mijn overleden kat.
- Ik ben snel boos.
- Ik eet te veel.
- Ik zie elke ochtend op tegen de dag.
- Mijn schuldgevoelens dat ik te weinig tijd heb voor mijn kinderen.
- De gedachte dat ik niet veel waard ben.
- Het gevoel van uitputting.
- Mijn onzekerheid.
- Dat ik te veel drink.
- Etc. etc.

Ik heb last van	Hoe lang al?

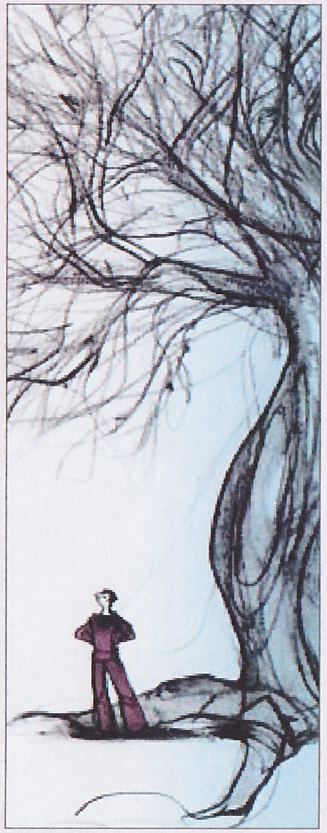

Je hebt al het psychisch leed uit je rugzak gehaald en voor je neer gezet. Het is de stand van zaken van dit moment voordat je aan deze cursus bent begonnen. En was het nu maar zo simpel dat je deze bagage naast je neer kon zetten en gewoon weg kon lopen Dan zou

Vorige stap | Volgende stap

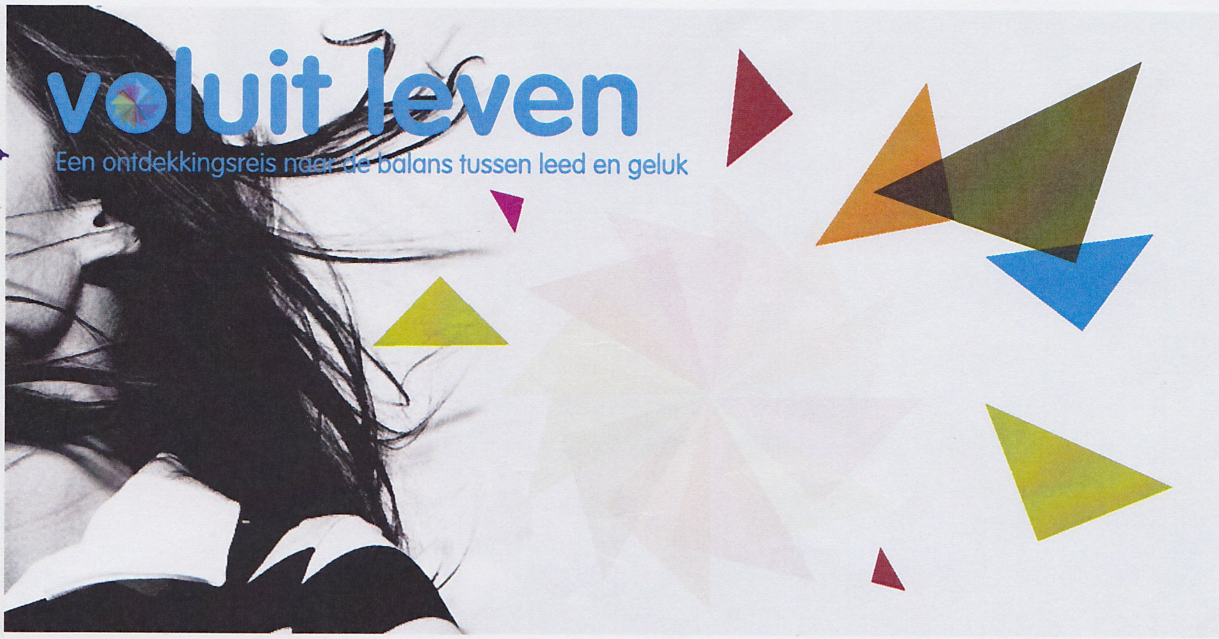

goede tekst →

naam | wachtwoord | login

informatie | aanmelden

Deze site gaat je niet gelukkig maken...

motiveert niet !!

Dat wil zeggen dat je hier niet leert hoe je een leven zonder teleurstelling, pijn, somberheid, angst, onzekerheid of twijfels kunt leiden.

Veel mensen ervaren hun leven als onbevredigend omdat zij last hebben van klachten als angst, somberheid, vermoeidheid en onzekerheid. Men denkt dat pas als deze klachten overwonnen zijn, men weer het leven kan leiden dat men graag wil leiden...

Voluit Leven laat je ervaren en inzien dat dit niet werkt. Het willen vermijden van psychische of lichamelijke pijn leidt tot een krampachtige poging om een half leven te leiden. Dit versterkt op den duur het psychisch leed. De methoden die in Voluit Leven worden aangereikt zijn gebaseerd op aanvaarding van psychisch leed en het vergroten van psychologische flexibiliteit. Aanvaarding maakt de weg vrij om met aandacht en vanuit je waarden te leven. Wanneer dit lukt ervaar je wel met de dag meer vrijheid en kwaliteit in je leven...

achtergrond bemoeilijk lezen v.d. tekst

Kleurige figuren – aantrekkelijk
foto v.d. persoon = overbodig

Koptekst voluit Leven prima
